# Supplementary material for: Are carbon emissions trading and green financial instruments synergistic? -Comprehensive quantitative research based on content analysis
Source: PLoS One. 2024 Mar 7;19(3):e0298601. doi: 10.1371/journal.pone.0298601 (PMC10919601; doi:10.1371/journal.pone.0298601)
Supplement: S1 Data — (ZIP) [file pone.0298601.s001.zip › Data/地级市Z_F工作报告.zip!/╕ú╜¿/╧├├┼╩╨/└┤╫╘╣1⁄2╓┌║┼í╛┬φ┐╦╩2╛▌═°í┐_2011.docx]

各位代表：

　　现在，我代表市人民政府向大会作政府工作报告，请连同《厦门市国民经济和社会发展第十二个五年规划纲要（草案）》一并审议，并请市政协委员和列席人员提出意见。

　　一、 2010年和“十一五”工作回顾

　　2010年，我们在中共厦门市委的领导下，全面贯彻党的十七大，十七届三中、四中、五中全会和胡锦涛总书记等中央领导来闽、来厦考察的讲话精神，深入贯彻科学发展观，打好“五大战役”，抓实“五个着力”，经济社会持续健康协调发展，较好地完成了全年各项任务。全年地区生产总值2053亿元，增长15.1%；规模以上工业总产值3670亿元，增长33.9%；全社会固定资产投资1010亿元，增长14.5%；财政总收入526亿元，增长16.5%，其中地方级财政收入289亿元，增长20.2%；社会消费品零售总额696亿元，增长21%；城镇居民人均可支配收入29253元，增长12%，农民人均纯收入10033元，增长9.6%；城镇登记失业率3.3%，居民消费价格指数103，人口自然增长率7.81‰；节能减排目标预计可以完成。

　　一年来的主要工作和成效是：

　　（一）改革开放更加深入，经济特区再创新优势

　　特区“示范窗口”作用更加凸显。厦门经济特区由厦门岛扩大到全市行政区域，为建设大特区拓展了发展空间，解决了“一市两法”等体制机制难题，已有50部特区法规和43部特区规章适用于新扩特区。国务院同意厦门建设两岸区域性金融服务中心，扩大金融改革试点，先行试验金融领域的重大改革措施。厦门成为国家创新型试点城市、服务业综合改革试点城市、创业型试点城市、低碳试点城市、首批三网融合试点城市、两岸食品物流合作试点城市、保险改革发展试验区、服务外包示范城市、流通领域现代物流示范城市、人口计生综合改革示范城市。行政审批制度、投融资体制、医药卫生体制、文化体制等领域改革不断推进，特区发展的动力活力进一步增强。

　　对外经贸交流进一步扩大。成功举办第二届世界投资论坛和第十四届投洽会，国家副主席习近平亲临会议并作主旨演讲，8位外国元首、54个国家（地区）的部长出席会议。厦门作为PNLG主席城市和秘书处所在地，为东亚海地区实施海岸带综合管理提供了范例。与香港贸发局签署合作备忘录，加强会展、金融、物流方面的战略合作。毕马威在厦设立分公司，至此全球四大知名会计师事务所齐聚厦门。全年实际利用外资22.6亿美元（历史可比口径）。厦门成为跨境贸易人民币结算试点城市，2217家企业获得试点资格，结算金额34.8亿元。外贸进出口总额570亿美元，增长31.7%，其中，出口353亿美元，增长27.7 %。

　　与荷兰皇家航空公司签约开辟厦门－阿姆斯特丹洲际客运航线，标志着厦门航线冲出亚洲、走向世界。成功举办第八届世界同安联谊大会。厦门国际马拉松赛、国际海洋周在海内外影响进一步扩大。厦门作为上海世博会城市最佳实践区综合案例首个亮相城市，受到广泛好评。

　　国际航运枢纽港建设加快推进。全面完成厦漳港口整合，厦门港由8个港区扩大到12个港区，新增港口岸线资源55公里、生产性泊位40个，开创了厦漳两市“资源共享、优势互补、管理高效”的良好局面。全港集装箱航线188条、月航班956班，分别比上年增加35条、140班。港口集装箱吞吐量582万标箱，位居世界港口19位，货物吞吐量1.39亿吨，比上年增长25.5%。海铁联运增长21.7%，集装箱异地货源出口比重超30%，厦门成为吸引异地货源出口和进口货物集散的重要外贸口岸。海沧保税港区一期封关运作。国际航运中心项目建成使用。与南澳洲弗林德斯港缔结友好港口关系，友好港口达18个。“雪龙号”从厦门港始发赴北极科学考察。厦门成为个案享受国际邮轮在我国沿海多港挂靠、两岸直航政策的首个城市，国际邮轮母港建设加快。

　　（二）加快转变发展方式，经济运行平稳健康协调

　　先进制造业实力增强。全年完成工业投资204亿元，增长23.6%。冠捷工业园、金桥卷烟技改等项目建成投产，46个重点产业振兴和技术改造项目进展顺利。全年规模以上工业增加值890亿元，增长24.5%。规模工业企业利润增长44.3%。工程机械等13条百亿以上制造业产业链实现产值2359亿元，占规模以上工业产值64.3%。平板显示产业链产值超600亿元，计算机与通信设备产业链产值接近600亿元。厦门火炬高新区成为国家首批新型工业化产业示范基地，也是福建省首个工业总产值超千亿的开发区。友达光电、戴尔（中国）、宸鸿科技、翔鹭石化4家企业产值超百亿元。

　　创新能力逐步增强。新认定高新技术企业100家，全市高新技术企业总数约占全省一半。新增1家国家级创新型试点企业、25家省级创新型试点企业。银祥集团建立了我市第一个依托企业的国家级重点实验室。新增万里石、金牌橱柜等15个中国驰名商标，专利授权量增长69%，知识产权转化成效显著。诊断试剂、基因工程药物等研究应用取得突破，戊肝疫苗的相关研究成果获国家技术发明二等奖。我市成为“国家知识产权工作示范城市”、“国家商标战略实施示范城市”和国家“十城千辆”节能与新能源汽车应用示范城市。

　　现代服务业发展加快。港口物流、金融保险等现代服务业产业群加速发展。观音山、湖里高新园等营运中心开始显现效益。浦发银行、华夏银行、赣州银行等一批金融机构落户厦门，君龙人寿、厦门银行等企业加快异地拓展。科华恒盛等6家企业成功上市。厦门石油交易中心挂牌成立。汽车物流中心动工建设，港丰物流等一批项目落户厦门。动漫节、石材展、药交会等重大展会成功举办。新增双龙潭等一批旅游景区和乡村旅游景点，来厦旅游人数达到3026万人次。建成龙山、集美集等一批文化创意产业园。华强文化科技产业基地、中移动手机动漫基地、中国数码港产业基地、中电信动漫运营中心落户厦门。SM新生活广场、五缘湾游艇帆船港及保税仓库等项目建成启用。

　　（三）城乡统筹稳步推进，岛内外一体化进程加快

　　中心城市框架实现新拓展。推进集美、翔安、同安、海沧四个新城建设，完成71平方公里核心区规划，杏林湾营运中心等178个总投资924亿元的拓展岛外项目全面展开。实施西郭、钟宅等旧城旧村改造，建设东部商务区，加快岛内老工业区“退二进三”步伐，万达城市综合体等一批提升岛内的项目扎实推进。翔安隧道，BRT二号线（同安线），厦漳、泉厦高速公路扩建工程建成通车，福厦高速铁路建成并开通动车组；海翔大道、厦门北站片区等重点项目顺利推进。海沧港区6个十万吨级、浏五店港区3个五万吨级泊位基本建成。莲花水库、九龙江北溪引水二期工程改造加快推进。市政设施建设不断完善，城市服务功能持续提升。

　　“三农”工作再创佳绩。加快推进统筹城乡发展综合配套改革，完成农村“三资”清理，发展农村社区股份合作经济，农村劳动力转移2万多人，农民人均纯收入继续稳居全省首位。农业龙头企业技术创新和品牌建设持续提升，食品工业实现产值235亿元，增长18.6%。都市现代农业加快发展，形成“一村一品”特色农业格局。农民专业合作社323家，带动农户4.9万户。开展汀溪、新圩小城镇改革发展试点，15个重点村旧村改造和9个老区山区村建设有序推进，全市自然村通达公路硬化率80%，行政村通客车率95%，25个山区村安全饮水工程全面完成。基本完成新一轮农村义务教育和公共卫生项目。

　　（四）前沿平台优势凸显，对台交流合作更加密切

　　经贸合作成效显著。做好ECFA签署的跟进工作，主动对接两岸产业“搭桥”计划，台盐大中华总部落户厦门，台资富邦财险公司在厦开业，台湾元富证券在厦设立代表处。成立首个两岸合作的呼叫服务中心、海峡物流合作发展中心。全年实际利用台资（含第三地）4亿美元。对台进出口总额62亿美元。大嶝对台小额商品交易市场进口台湾商品增长48.5%。我市台湾水果进口量增长39.6%，居大陆各口岸首位。厦门国贸等企业赴台设立分支机构。市经贸文化交流考察团赴台“走亲访友做生意”取得实际效果。台交会、海峡两岸图书交易会圆满成功。

　　交流交往更加密切。成功举办第二届海峡论坛，全国政协主席贾庆林莅会演讲，1万多名台湾各界人士参加。国家有关部委在厦设立对台科技、农业、体育、新闻出版和会计交流合作基地。率先获准为部分公务人员办理“一年有效、多次往返”签注。厦门成为货运直航包机新增航点，开通了海西首条对台全货机航线，每周空中往返客运航班达60班。厦金“小三通”每日航班增至36班并实现行李双向托运，全年进出旅客达127万人次。经厦门赴台旅游的大陆游客达到16.5万人次，增长84.5%。成功举办海峡两岸文博会、民间艺术节、龙舟赛、海上联合搜救演练、海峡杯帆船赛、厦金海峡横渡、郑成功文化节等重要活动。保生大帝神像首次赴台巡游。厦门卫视在境外落地取得新进展。厦台合作的歌仔戏《蝴蝶之恋》获得文华大奖。厦台合拍的电视剧《神医大道公》深受观众好评。大陆首个闽台合作的数字创意学院落户厦门理工学院。

　　（五）民生保障更加有力，和谐厦门建设有效推进

　　社会保障不断加强。构建和谐劳动关系，倡导“感动人的管理”，全年新增就业19万人。建立城乡一体化基本医疗保险制度，实现养老保险城乡全覆盖。企业退休人员基本养老金人均月增180元，达到1814元。6万多名被征地人员办理了养老保险，其中4.2万人办理退养手续，月均退养金达725元。城乡居民养老保险登记参保14万人，60周岁以上居民直接享受每月200元基础养老金。政府出资为全市农村住房办理统一保险，为全市市民办理自然灾害公众责任险，为全市孤儿办理重大疾病公益保险。对低收入居民实行动态生活补贴。设立70个社区居家养老服务中心（站），建成启用社会福利中心。实施户籍新政，放宽户籍准入。全市财政投入4.2亿元补贴公共交通。加强市场监管，主要农产品、农副食品和公用行业价格总体保持稳定。完成12件为民办实事项目。援建四川彭州工作圆满完成，对口支援新疆、西藏、宁夏、南平等工作成效显著。

　　社会事业全面发展。全市财政用于社会事业投入68.7亿元，增长14.9%。新建7个义务教育项目，新增学位1.1万个。进城务工人员子女在公办校接受义务教育比例达68.8%，就读公办初中比例达82.2%。完成185幢中小学校舍抗震加固安全工程。为全市中小学校和幼儿园配备保安。全面实施国家基本药物制度。启动公立医院改革，完善公立三甲医院与基层医疗卫生机构对接机制。行政村卫生所覆盖率达到100%，社区卫生服务中心标准化建设全面完成。对农村孕产妇住院分娩给予人均400元补助。计生政策符合率达97.15%，6个区全部成为全国计划生育优质服务先进单位。小白鹭艺术中心剧场等文化设施投入使用，设立国家数字图书馆厦门分馆。成功举办国际钢琴比赛等一批重大文化赛事。荣获全国文明城市指数测评第一名。我市运动员在广州亚运会和亚残会上取得优异成绩。

　　人居环境持续改善。开口改造高集、集杏海堤，海洋生态逐步修复。新建12个社区公园，新增园林绿地700公顷，城市主干道和重要节点景观整治取得成效。完成天地湖污水截流工程，扩建石胄头、同安污水处理厂，城市污水集中处理率达86.5%。城乡生活垃圾无害化处理率达97%。重点整治机动车尾气、工业废气、油烟污染，空气质量优良率达98.2%。重视改善交通，新建一批公共停车场和公交站点，荣获全国“畅通工程”模范城市称号。

　　“平安厦门”深入推进。加强社会治安综合治理，深入开展专项整治，信访、基层司法调解和多元化纠纷调处工作不断加强，群众安全感和社会治安满意率位居全省前列。实施重点行业领域安全生产专项整治，强化重大危险源监控，完成省下达的安全生产责任目标。加强防空防灾和应急指挥平台建设，荣获全国人民防空先进城市。国防动员、优抚安置、双拥共建、民兵预备役、海防等工作取得新进展。

　　（六）加强自身建设，政府服务水平进一步提高

　　制定加强法治政府建设实施意见，强化行政执法监督，规范行政自由裁量权，依法行政水平进一步提高。“五五”普法顺利完成。提请市人大常委会审议法规草案4件，制定、修订政府规章14件。自觉接受人大及其常委会监督，依法执行人大决定决议，向人大及其常委会报告工作7次46项，办理人大代表议案8件、建议220件，配合人大常委会执法检查、人大代表视察及专项调研80多场次。支持人民政协履行政治协商、民主监督、参政议政职能，定期通报政情，办理政协提案506件。加强廉政建设，强化审计监督、专项监察，机关效能建设和纠风工作取得实效。

　　各位代表！2010年的工作为“十一五”画上了圆满的句号。过去的五年，我们紧紧把握海西建设的重大机遇，成功应对国际金融危机的严重冲击，有效克服发展中的各种困难，保持了经济社会又好又快发展的良好势头。一是综合经济实力大幅提升。地区生产总值年均增长14.1%，财政总收入年均增长20.9%，地方级财政收入年均增长24.1%，社会消费品零售总额年均增长19.3%。人均地区生产总值居副省级城市前列，万元生产总值能耗保持全国领先水平。二是产业结构更加优化。三次产业结构从2005年的2:55.6:42.4调整为2010年的1.1:50:48.9，第三产业增加值年均增速15.8%，每平方公里创造地区生产总值1.31亿元、财政收入3344万元，财政总收入占地区生产总值比重达25.6%。建成视听通讯、钨材料、软件、半导体照明、电力电器等5个国家特色产业基地，高新技术企业达524家。三是中心城市地位更加凸显。五年累计完成固定资产投资4413亿元，是“十五”期间3.3倍，其中岛外投资占56.1%。建成同安工业集中区、集美机械工业集中区等产业园区，环东海域、五缘湾、杏林湾、观音山等片区开发快速推进，发展空间进一步拓展。城市快速交通网络基本形成，以港口、高铁、航空和高速公路为主体的综合交通枢纽基本建立，对外辐射能力进一步增强。金融商贸、旅游会展、航运物流等设施不断完善，中心城市服务功能持续提升。四是改革开放迸发新活力。整合重组23家国企集团，国有资产增加1501亿元，年均递增20.8%。厦漳港口整合和口岸管理一体化有序推进。保障性住房建设管理模式成为全国住房制度改革的“蓝本”。在行政管理、投资融资、社会事业、公共资源配置等领域的改革不断深化。五年实际利用外资76亿美元，新引进23家世界500强企业。国际友城增至15个，接待来访外宾团组958批8122人次，国际知名度进一步提升。五是对台交流合作取得新突破。五年实际利用台资28.5亿美元，对台进出口贸易额194亿美元。率先成为“大三通”的口岸之一，成为两岸包机直航点、海上直航口岸、通邮封发局。率先试点开展在厦暂住人员赴金门旅游。厦金“小三通”航线成为两岸人员往来的“黄金通道”。引进大陆第一家台资保险法人机构、台资全资保险机构。厦门银行成为台湾金融机构借道第三地入股大陆银行首例。大陆最大的台资合作医院落户厦门。率先开展在厦台湾居民技术职务任职资格评审试点。率先在事业单位招聘台湾专门人才。打造了海峡两岸文博会、图书交易会、农渔业论坛等一批交流合作平台。六是生态文明建设扎实推进。实施“蓝天、碧水、绿色、宁静、洁净”工程，综合整治筼筜湖、五缘湾、杏林湾和环东海域。万元GDP综合能耗约为全国平均水平的一半，主要污染物排放量超额完成“十一五”减排任务，被授予全国“节水型城市”和“节能减排二十佳城市”。森林覆盖率42.8%，人均绿地近30平方米，城市自然景观和人居环境持续改善。七是人民生活品质稳步提升。城镇居民人均可支配收入和农民人均纯收入年均递增12.3%和10%。最低工资标准和最低生活保障线分别提高44.4%和23%。成为全国推进义务教育均衡发展工作先进地区。在全国率先实现“全民医保”，首创“建筑工地农民工大病保险”。率先建立市民健康信息系统，荣获第五届中国地方政府创新奖。建成文化艺术中心等一批重大社会事业项目，文化体育惠民工程取得新实效。农村劳动力转移就业超过10万人。实施旧村和老区山区村改造建设，开展农村家园清洁行动，农村面貌变化大、提升快。群众对社会治安满意率持续位居全省前列。2008年在首批全国文明城市复查考评中，总分再次名列第一。

　　各位代表！这些成绩的取得，是在省委、省政府和市委的正确领导下，全市人民齐心协力、艰苦奋斗的结果。在此，我谨代表市人民政府，向全市人民，向给予政府工作大力支持与有效监督的人大代表和政协委员、各民主党派、工商联、各人民团体、无党派人士、离退休的老同志和社会各界人士，向中央和省驻厦单位、驻厦部队、武警官兵和公安干警，向来厦的投资者和劳动者，向关心支持厦门发展的港澳同胞、台湾同胞、海外侨胞和国际友人，表示衷心的感谢和崇高的敬意！

　　同时，我们也清醒地看到，当前发展还面临不少困难和问题，主要是：经济发展方式转变任务艰巨，产业结构调整升级难度加大；投资总量偏小和投资结构不尽合理，发展后劲不足；经济腹地、市场空间拓展有限；自主创新能力不足，创新型人才和实用型技工短缺；违法建设查处机制不够健全，征地拆迁工作有待进一步加强；人民币升值、人工成本上升和原材料价格上涨等对进出口影响较大；改革的任务还很艰巨，一些体制性障碍亟待破解；社会矛盾不断增多，社会管理创新不足；一些部门回避矛盾、推诿扯皮的现象依然存在，部分政府工作人员积极进取、敢于负责的责任感不强。我们要正视这些问题，认真加以解决。

　　二、 “十二五”时期的展望

　　“十二五”是加快转变经济发展方式、推动跨越发展的关键时期，是厦门经济社会发展大有可为的战略机遇期。提请大会审议的《厦门市国民经济和社会发展第十二个五年规划纲要（草案）》（以下简称《规划（草案）》），总结“十一五”规划实施情况，分析“十二五”面临的机遇和挑战，提出今后五年经济社会的发展目标、战略任务和保障措施。

　　根据党的十七届五中全会、省委八届十次全会和市委十届十三次全会的部署，我市“十二五”发展的指导思想是：高举中国特色社会主义伟大旗帜，以邓小平理论和“三个代表”重要思想为指导，深入贯彻落实科学发展观，以厦门经济特区范围扩大到全市为新起点，以科学发展为主题，以加快转变经济发展方式为主线，坚持一体化提效提速、二三产做强做大、内外需并重并进、惠民生优先优质，发挥好“排头兵、示范窗口、龙头带动”作用，加快推进我市科学发展新跨越。

　　《规划（草案）》围绕主题、突出主线，提出经济发展、民生发展、科教发展和可持续发展四大类21项主要目标，其中约束性3项、预期性18项。明确到2015年，全市生产总值、财政总收入、高新技术产业产值比2010年翻一番以上，全社会固定资产投资总额、民生和社会事业投入比“十一五”翻一番以上；大力打造“创新厦门”、“宜居厦门”、“平安厦门”、“文明厦门”和“幸福厦门”。上述目标体现质量、效益、结构、速度的统一，体现经济发展和民生改善的统一，体现量力而行和尽力而为的统一，经过全市人民共同努力是可以实现的。

　　今后五年经济社会发展的战略任务主要有以下十个方面：

　　（一）全力推进岛内外一体化，在统筹城乡发展上实现新突破。按照“规划一体化、基础设施建设一体化、基本公共服务一体化”的总体要求，全面拓展岛外，优化提升岛内，推动城市建设和产业发展双轮驱动，建成岛外71平方公里新城核心区，城市建成区面积超过300平方公里，城市化率达85%，城市综合实力和核心竞争力显著提升，基本形成岛内外一体化、岛外各组团独具特色又相互协调的城市新格局。

　　（二）坚持二三产共推进，在优化产业结构和壮大产业规模上实现新突破。加强科技创新与品牌建设，推动信息化与工业化、制造业与服务业互相融合，做强做大支柱产业，重点培育战略性新兴产业，改造提升传统优势产业，打造海峡西岸先进制造业基地和最具竞争力的现代服务业聚集区。力争五年内形成若干个500亿元乃至1000亿元以上产值的产业链（群），打造若干个500亿元乃至1000亿元以上年销售（营业）收入的大企业集团。

　　（三）坚持内外需齐拓展，在全方位大开放上实现新突破。充分利用国际国内两个市场、两种资源，加快外经贸增长方式转变，提高利用外资水平，努力扩大消费需求，优化投资结构，加强区域协作，推动经济发展方式向内需与外需并重、消费与投资并重转变，全面提升开放型经济发展水平。

　　（四）始终突出对台先行先试，在对台交流合作上实现新突破。围绕建设两岸交流合作先行区，全面深化对台经贸合作，建设两岸货物贸易的重要枢纽和产业对接集中区。拓展两岸直接往来的范围和层次，提高两岸直接往来管理和服务水平，建设两岸直接往来的综合枢纽和“黄金通道”。创新对台交流合作机制，建设两岸交流交往最活跃的平台。充分发挥“五缘”优势，建设两岸同胞融合示范区。

　　（五）加大改革攻坚力度，在体制机制创新上实现新突破。加快重点领域和关键环节改革，积极争取设立国家综合改革试验区。以服务业综合改革和财政体制、投融资体制和国有企业等领域改革为重点，建立促进转变经济发展方式的长效机制。以建立健全基本公共服务体系保障机制为重点，加快推进教育、卫生、文化等社会领域改革。以统一岛内外管理体制为重点，加快构建有利于岛内外一体化的体制机制。以建设法治政府和服务型政府为重点，深化行政管理体制改革。

　　（六）完善基础设施保障体系，在中心城市建设上实现新突破。围绕构建海陆空立体交通运输体系，完善水电气供应保障以及防灾减灾体系，增强城市承载能力和综合服务功能。力争到2015年港口货物吞吐量突破2亿吨、集装箱吞吐量突破1000万标箱，形成国际集装箱中转中心，打造海峡西岸航运物流中心和国际航运枢纽港。完成厦门高崎机场三期扩建，力争动工建设翔安国际机场，实现年旅客吞吐量2300万人次、货邮吞吐量达30万吨，打造东南沿海重要的国际干线机场和区域性航空枢纽港。打造东南沿海铁路交通综合枢纽和全国重要公路主枢纽城市，构筑厦漳泉同城化快速通道，完善市政交通路网构架，形成闽西南“1小时交通圈”和市域内“半小时生活圈”。

　　（七）加快资源节约型、环境友好型社会建设，在生态文明建设上实现新突破。加快国家低碳试点城市建设，推进产业发展、城市建设、生活方式低碳化。强化节能减排，发展循环经济，加强生态环境保护，提升生态文明水平，严格落实节能减排目标责任制，继续保持单位生产总值能耗、碳排放强度、主要污染物排放量等指标居全国、全省领先，打造“宜居厦门”。

　　（八）实施科教兴市和人才强市战略，在创新型城市建设上实现新突破。加大自主创新力度，优先发展教育，实施人才强市战略，基本建成创新体系完善、创新人才荟萃、创业投资活跃、综合环境优良的国家创新型城市，打造“创新厦门”。到2015年高新技术产业产值翻一番以上，全市科技研发投入占地区生产总值4%，建成50个科技创新公共平台和30个科技园区、专业孵化器。

　　（九）着力惠民生促和谐，在基本公共服务保障上实现新突破。坚持保基本、广覆盖、可持续原则，加快推进岛内外和城乡基本公共服务均等化，基本公共服务整体水平位居全国前列，社会更加和谐稳定，人民幸福指数不断提高，打造“幸福厦门”。

　　（十）大力推进文化强市建设，在增强城市软实力上实现新突破。全面实施文明品牌工程，健全文明城市创建长效机制，打造“文明厦门”。全面实施平安创建工程，提升社会管理水平，打造“平安厦门”。完善公共文化服务体系，加快文化产业发展，打造海峡两岸文化合作交流的重要基地，建设具有鲜明特色的国际知名文化城市。

　　三、 2011年工作建议

　　2011年是“十二五”开局之年，也是厦门经济特区建设30周年。我们要全面贯彻落实省委、省政府和市委的决策部署，继续打好“五大战役”、大干开局之年。经济社会发展主要预期目标为：地区生产总值增长15%，规模以上工业总产值增长17%，全社会固定资产投资增长25%，外贸进出口增长14%，实际利用外资与上年基本持平，地方级财政收入增长16.3%，城镇居民人均可支配收入和农民人均纯收入均增长12%，城镇登记失业率控制在4.5%以内，居民消费价格指数104左右，人口自然增长率控制在9.5‰以内，完成国家和省下达的节能降耗指标任务。

　　实现上述目标，必须扎实做好以下工作：

　　（一）推进产业结构优化升级，加快发展方式转变

　　大力发展先进制造业。着力壮大一批产业群、落实一批大项目、培育一批大企业、打造一批大基地、创新一批大品牌。做大做强电子、机械支柱产业，建成一批产业龙头项目和高新技术产业项目，培育百亿企业集团和千亿产业链。抓好天马面板、金财液晶面板、宸鸿科技、厦工挖掘机等新建扩建项目，加快建设太古飞机六期、产业技术研究院等续建项目，积极推进抽水蓄能电站等重点前期项目，确保一批重大项目按计划顺利启动。支持企业技术改造，增强新产品开发和品牌创建能力。完善工业园区基础设施和服务配套，营造有利于大项目生成落地的环境条件。

　　大力提升现代服务业。围绕服务业发展提速、比重提高、水平提升，培育壮大航运物流、旅游会展、金融与商务、软件与信息服务等重点发展的现代服务业。推动优质企业在境内外上市融资和后续融资，推进火炬高新区进入“新三板”扩容试点，加快建设两岸区域性金融服务中心。加快前场铁路大型货场建设。积极拓展内陆腹地，大力发展“陆地港”、国际中转业务和第三方物流。加大“海峡旅游”品牌宣传，推进国际邮轮母港建设，推动游艇帆船、高端医疗等产业发展，提升鼓浪屿等景区的服务品质，推出一批新的旅游景点，培育开发温泉游、生态游等旅游产品，开工竣工一批高星级酒店，提升厦门旅游目的地竞争力。大力发展信息数据处理、呼叫中心等业务流程外包，积极承接软件开发及设计等研发流程外包。加快建设世贸海峡大厦等营运中心，积极引进国内外大型企业集团总部、区域总部、结算中心、销售中心。强化会议目的地宣传营销，创办航空维修和服务展、网络购货展等专业展会，做大做强品牌展。

　　大力培育战略性新兴产业。进一步明确产业发展重点，强化政策引导，加快项目实施。新一代信息技术方面，重点发展新一代移动通信、云计算技术及服务、高端软件和集成电路设计等产业，推动“智慧厦门”产业基地等一批项目建成投入运营。生物与新医药方面，重点发展诊断试剂、基因工程药物、海洋生物医药等产业，建设厦门生物产业基地。新材料方面，重点发展新能源电池材料、太阳能电池、特种金属材料等产业，建设厦门新材料研究院和产业化基地。节能环保方面，重点发展高效节能装备、节能照明产品、废水膜治理回用等产业，推进国家“十城万盏”试点城市、“十城千辆”节能与新能源汽车应用示范工程和“金太阳示范工程”建设。文化创意方面，重点发展创意设计、影视动画、文化旅游、数字内容四大产业集群，推进华强文化科技产业基地、湖里文创园等重大项目建设。海洋高新产业方面，重点发展海水综合利用、海洋精细化工、海洋能源利用等产业，加快建设厦门国家海洋研究中心。

　　大力增强自主创新能力。以公共科技服务平台、重大科技项目建设为支撑，全面推进国家创新型城市建设。加快国家科技成果转化服务（厦门）示范基地、科技创新园、火炬高新区创新型科技园建设，推动北斗卫星综合移动终端应用开发、闽台诊断产品创新创业园等重大平台建设，支持筹建中科院技术转移机构、海峡农业技术研究院。加强院地、市校、两岸及国际科技合作，发挥“6·18”等平台作用，促进科技成果转化。积极引进中船重工七二五所、中电三十所等央企研发机构，支持企业建设国家工程技术研究中心和重点实验室。深入实施标准化战略和质量强市战略，建立质量发展推进机制。加强知识产权创造、运用和保护。大力实施《厦门市中长期人才发展规划纲要》，积极对接国家“千人计划”，实施人才“双百计划”。继续实施教育服务产业发展行动计划，加大紧缺技能型人才培训培养支持力度，完善人才评价办法和激励机制，吸引人才、留住人才、用好人才。

　　（二）拓展岛外提升岛内，加快岛内外一体化进程

　　按照“全域厦门”理念和“四高”要求，进一步做好大特区的城市规划、产业布局和土地利用工作。一是岛外新城建设要有新进展。围绕“抓规模、出形象、保重点”，加快新城启动区开发，推进一批标志性工程建设，抓紧建设学校、医院、酒店、商业网点等公共配套设施，尤其是抓紧建设区位较好、规模较大、配套完善的限价商品房小区，尽快聚集人气，打造新的城市亮点。二是岛内功能优化提升要有新作为。遵循“保持风貌、保持特色，降低开发强度、降低建设密度，提升环境品质、提升城市功能”的原则，完善岛内规划，优化环境品质和城市功能，引进高端项目，促进产业升级，加快建设金融服务中心、商务营运中心、旅游会展中心、消费购物中心。三是岛内外产业对接、政策体制一体化要有新突破。加快城市中心区“退二进三”步伐，为产业结构优化腾出空间。逐步统一岛内外民生福利标准，引导城市优质教育、卫生、文化资源向农村延伸，提升岛外公共服务水平。

　　（三）拓展内需市场，发挥消费投资出口协调拉动作用

　　积极扩大内需。增加政府支出用于改善和扩大消费的比重，增强居民消费能力。优化商业网点布局，抓好商贸聚集区、商业特色街和大型专业批发市场建设，大力发展品牌连锁经营，积极发展电子商务、网络购物等新型消费业态。丰富消费品种，扩大文化娱乐、体育健身、休闲旅游、信息通讯等消费，适度引导高端消费。落实国家扩大消费的优惠政策，把握高铁动车带来的新商机，营造良好消费环境，促进扩大消费。

　　优化投资结构。注重提高工业和服务业投资比重，重点抓好新兴产业、重大片区、基础设施、民生保障、生态环境等建设投资。鼓励社会资本进入旧城旧村改造、基础设施建设和社会事业等领域。抓好173个重点建设项目，完成年度投资476亿元。围绕产业发展、新城建设，策划生成一批中长期发展项目。加快轨道交通、翔安国际机场等项目前期工作。优先保证在建和续建项目的资金需求，有序启动“十二五”规划重大项目建设。严格执行投资项目用地、节能、环保、安全等准入标准，提高投资质量和效益。

　　转变外贸增长方式。优化贸易结构，创新商业模式，大力发展服务贸易。培植壮大一批出口基地、创新一批自主出口品牌、培育一批流通型外贸企业和大型民营出口企业，争取设立中国（厦门）国际贸易中心。做大与东盟的贸易规模，巩固扩大纺织服装、轻工产品等市场份额，扩大客车、钨制品、游艇等优势产品出口，鼓励科技含量和附加值高的机电产品、高新技术产品出口。积极拓展进口，力争成为若干大宗商品进口集散地和分拨地。推进大通关和电子口岸建设，加强口岸通关、市场预测预警、贸易摩擦应对指导等服务体系建设。鼓励有条件的企业并购境外知名品牌、营销网络和研发机构，在全球范围内开展资源、供应链和价值链整合。

　　（四）深化改革开放，不断提升开放型经济水平

　　推进体制机制创新。做好新一轮政府机构改革，进一步理顺部门职责权限，完善市对区管理体制。稳步推进事业单位和收入分配改革。加快文化体制改革。深化国企股份制改革，完善国企法人治理机构，加快国有资本战略性结构调整。鼓励国企到异地拓展业务。推进投融资体制改革，加强资源要素市场化配置。落实促进中小企业发展的政策措施，提升民营经济发展水平。稳步实施油气水电等资源性产品价格改革，推进鼓励类服务业用电、用水、用气、用热与工业基本同价。

　　提高招商引资效益。充分发挥产业园区招商引资的载体作用，强化引进项目的产值、税收及科技含量，优化引资结构，提升引资质量。依托现有先进制造业和现代服务业产业链（群），加强项目策划生成和包装，着力引进带动力强的龙头型、效益型项目。加强与央企、省企和大型民企的战略合作。加强与国内外著名投资咨询中介机构合作，吸引世界500强企业、跨国公司、创投企业、风险投资者来厦布局。发挥闽商、各类商协会和华侨华人社团作用，加强日常联络和项目推介，提高境内外招商成效。加强区域协作，做好对口援建和帮扶工作，密切闽西南五市和闽粤赣十三市的合作。

　　加强对外交流合作。加快领事馆区二期建设，推动更多外国领事机构落户厦门。加强城市营销，推动爱乐乐团、小白鹭民间舞团等文化品牌走向世界，提高厦门的国际知名度和美誉度。加强与国际组织的合作，提高投洽会、石材展等展会的国际化程度，争取举办更多的大型国际性、区域性会议和赛事。加强与国际友好城市、友好港口的实质性交流，开展互利互惠合作。推进厦漳同城化，加快厦漳泉大都市区建设。

　　（五）深化两岸经济文化交流，更好发挥前沿平台作用

　　着力产业对接。深入推进对台产业的对接和整合。拓宽两岸跨境贸易人民币结算渠道，探索引进台湾兴柜交易机制，推动设立柜台交易市场。争取率先落实ECFA早收清单，推动更多先行先试内容列入ECFA补充协议，争取在贸易投资便利化、台湾服务业市场准入等方面先行试验。做大做强大嶝对台小额商品交易市场。推动我市企业赴台投资、融资。

　　着力便利往来。推动厦门与台湾电子口岸的互联互通和信息共享。加强对台旅游合作，共推双向旅游线路，争取实施厦门居民赴台个人游。推动厦金海底通信光缆铺设工程建设。争取设立对台通信业务汇接局。推进对台邮件中转地和邮件中心建设。加快建设五通码头改扩建二期工程，推动厦门—金门行李双向直挂，吸引更多大陆居民和台湾民众循厦金“小三通”往返两岸。争取进一步开放航权、优化航路、增辟航线、增加航班，加快两岸海空运输综合枢纽建设步伐。

　　着力载体建设。精心筹办第三届海峡论坛。继续办好台交会、文博会、农渔业论坛等涉台盛会。充分发挥现有对台交流合作基地作用，争取更多国家部委来厦设立对台交流合作基地。深化厦门与台中两市基层交流。拓展厦台社会各界的对口交流和互动合作。全面提升厦台之间文教卫体、民俗宗教等方面交流的水平和实效。

　　（六）统筹城乡发展，加快农村城市化进程

　　拓宽农民稳定增收渠道。认真落实促进农村劳动力转移就业的各项优惠政策，广辟适合农民就业的公益性岗位，积极发展农村现代物业配套项目，支持以创业带动就业，争取转移就业1.5万人。深化统筹城乡发展综合配套改革，稳妥推进“村改居”社区集体资产改制，大力发展农村社区股份合作经济组织，探索建立“村改居”社区基层治理新机制。

　　提升现代都市农业水平。坚持“两头在厦、中间在外”的发展思路，加强科技研发，实施品牌战略，做大做强农业龙头企业。鼓励发展种苗业、都市休闲农渔业，建设对台农渔业新技术、新品种引进合作交流基地，发展一批高效益的精致设施农业。因地制宜发展乡村旅游和森林人家，建设一批集园艺、科普、观光为一体的生态休闲农业基地。

　　提高农村公共服务水平。以小城镇改革发展为载体，实现新农村与新城区建设的协调推进。推进移民造福工程，加强老区山区村建设，加快地质灾害点、重点水源保护区居民搬迁。强化以水利为主要内容的农业基础设施建设，启动大中型水闸除险加固。继续实施旧村改造和新村建设，加快完善农村道路、供水、通信、垃圾及污水处理等设施，将农村环境卫生逐步纳入市政管理体系，改善农村生产生活环境。

　　（七）加快经济社会协调发展，促进社会事业全面进步

　　促进教育加快发展。实施中长期教育改革发展规划纲要。优化基础教育资源配置，加快城乡教育均衡发展。试行“十年义务教育”。推进义务教育学校标准化建设。继续实施校舍安全工程。免除义务教育阶段学生的课本费和簿籍费。加强学前教育，新建一批公办幼儿园。提高进城务工人员随迁子女就读公办中小学比例。扶持民办教育健康发展。继续共建厦大、集大和华大。加快厦大翔安校区、城市职业学院轻工分院、理工学院三期等建设。大力发展职业教育。提升华文教育水平，扩大教育国际合作与交流。

　　加快医药卫生事业发展。推进翔安医院、五缘湾医疗中心、中山医院内科病房楼、厦门眼科中心扩建工程、口腔医院迁建项目建设，启动海沧医院二期等项目。加强公共卫生服务体系建设，加快公立医院改革，推广基本药物制度。改造提升公立基层医疗卫生服务机构，加快村卫生所标准化建设，提高乡村医生津贴补助，解决基层医疗卫生人才短缺问题，提升基层医疗机构服务水平。继续稳定低生育水平。做好迎接国家卫生城市复核工作。

　　重视文化体育事业发展。加快闽南戏剧艺术剧院、集美书城、诚毅科技馆、市运动训练中心、青少年校外活动中心等项目建设，启动厦门大剧院前期工作，新建一批文化馆、图书馆、社区文化广场和体育健身场所。推动文化创意产业园建设，争取设立国家级数字出版基地。推进闽南文化生态保护实验区建设。筹建中国非物质文化遗产博物馆。做好鼓浪屿申报世界文化遗产工作。办好国际马拉松赛、海峡杯帆船赛、世界剧协大会、中国诗歌节等重大活动。

　　（八）提升民生保障水平，促进和谐社会建设

　　进一步扩大就业。加大就业援助力度，完善就业创业服务体系，扶持发展家庭服务业，对劳动密集型小企业实施小额贷款担保支持，鼓励机关、企事业单位开辟公益性岗位，力争新增就业18万人。加强劳动仲裁和劳动保障执法监察，维护劳动者合法权益。

　　完善社会保障体系。制定流动就业人员基本医疗保险关系转移接续办法，逐步将符合条件的农村基层医疗卫生机构纳入基本医疗保险定点范围，方便参保农民就近就医。建立基本养老金、失业保险和最低工资标准动态调整机制，提高低保标准。大力发展社会福利事业，完善社会救助体系，支持慈善公益事业，做好扶老救孤、助残济困工作。

　　推进民生项目建设。整顿和规范房地产市场秩序，遏制投机性购房，启动公共租赁房和限价商品房建设，新建保障性住房1万套以上。加强“菜篮子”工程建设，增加粮食和重要农产品储备，确保农副产品市场供应和价格稳定。继续改进改善交通，加快综合客运枢纽、公交场站、公共停车场和交通集散配套工程建设，开展城市慢行系统建设，优化公交线路，提升客运服务质量。开工建设长泰枋洋人饮用水第二水源枢纽工程。推进汀溪水库群、同安东西溪下游河道等综合整治。实施城市精细化管理，加大环境污染综合整治力度。做好厦门岛与翔安供水干管互通改造。加快垃圾焚烧发电厂、东部固废处理中心等一批环卫设施建设，加大环岛路、松柏湖截污治理力度。推进天马山殡仪馆、金山养老院、市救助管理站等项目建设。

　　深化“平安厦门”建设。健全社会治安防控体系，依法打击各类违法犯罪活动，保障人民生命财产安全。推进社会管理创新，建立健全重大项目社会稳定风险评估机制，完善社会矛盾纠纷排查、调处机制，注重来信来访的合法合理解决。加强重点污染源、重大危险源监管和工业固体、危险废物处置，强化消防安全和道路交通安全管理，杜绝重大安全生产事故。推进防空防灾一体化建设，完善应急管理机制，提高应急处置能力。加强食品药品市场监管，确保市民食品和用药安全。

　　提升城市文明程度。创建文明城市，有利于改善人民生活，有利于提升市民文明素质，有利于增强城市综合竞争力。今年是第三届全国文明城市的总评年，我们要树立必胜信念，保持昂扬的精神面貌，举全市之力争创“三连冠”。

　　四、 加强自身建设，不断提升行政能力和服务水平

　　厦门经济特区有今天的良好局面，最重要的是解放思想、先行先试、开拓进取、务求实效。我们要站在经济特区建设30周年新的历史起点上，努力为未来探新路、谋新篇、打基础、争先行。

　　建设开拓型政府。不断强化特区意识、创新意识和责任意识，勇立潮头、勇挑重担、勇于进取，不自满、不松懈、不停步，在先行先试、开拓创新中抢占新一轮发展的制高点，在敢闯敢试、顽强拼搏中赢得更大的竞争优势。用足用好已有的政策，积极争取具有全局意义的新政策，大胆推出促进发展的新举措，不断增创经济特区发展新优势。

　　建设服务型政府。强化政府公共服务职责，加快政府职能转变。完善集中行政审批制度，健全政府服务企业、服务社会、服务群众长效机制，进一步营造良好的政务环境。深化机关效能建设，增强比学赶超的紧迫感和使命感，确保令行禁止、政令畅通，努力形成优质、高效的良好政风。

　　建设法治型政府。全面做好人大代表议案、建议和政协委员提案办理工作，自觉接受人大及其常委会的法律监督、工作监督和政协的民主监督，认真听取人大代表、政协委员和人民群众的意见、建议。坚持依法行政，强化执法监督，开展相对集中行政复议审理和行政复议委员会试点工作，拓展政务公开范围，建设规范透明的政务环境。

　　建设学习型政府。世界潮流浩浩荡荡，社会变化日新月异，城市发展百舸争流。厦门要在激烈的竞争中立于不败之地，要加快国际化、现代化进程，需要一批具有世界眼光和战略思维的领导干部，需要一支具有较高素质和充满活力的公务员队伍。为此，要注重理论学习，紧密联系实际，优化知识结构，提高工作水平，增强解决实际问题的能力。

　　建设廉洁型政府。严格执行党风廉政建设规定，扎实推进政府系统反腐倡廉工作，严肃查处各类腐败案件，纠正损害群众利益的不正之风。加强审计监督，强化政务督查。认真清理“小金库”，深入开展创建节约型机关活动，大力弘扬艰苦奋斗、勤俭办事的优良传统，树立风正气顺、清正廉洁的良好形象。

　　各位代表！让我们更加紧密地团结在以胡锦涛同志为总书记的党中央周围，高举邓小平理论和“三个代表”重要思想伟大旗帜，全面贯彻落实科学发展观，在市委的领导下，团结带领全市人民，开拓进取，扎实工作，以优异的成绩迎接中国共产党成立90周年！
